# The Tupange Project in Kenya: A Multifaceted Approach to Increasing Use of Long-Acting Reversible Contraceptives

**DOI:** 10.9745/GHSP-D-15-00306

**Published:** 2016-08-11

**Authors:** Michael Muthamia, Kenneth Owino, Paul Nyachae, Margaret Kilonzo, Mercy Kamau, Jane Otai, Mark Kabue, Nelson Keyonzo

**Affiliations:** a Jhpiego, an affiliate of Johns Hopkins University, Nairobi, Kenya

## Abstract

**Background:**

Long-acting reversible contraceptives (LARCs) are safe and highly effective, and they have higher continuation rates than short-acting methods. Because only a small percentage of sexually active women in Kenya use LARCs, the Tupange project implemented a multifaceted approach to increase uptake of LARCs, particularly among the urban poor. The project included on-site mentoring, whole-site orientation, commodity security, quality improvement, and multiple demand-promotion and service-provision strategies, in the context of wide method choice. We report on activities in Nairobi between July 2011 and December 2014, the project implementation period.

**Methods:**

We used a household longitudinal survey of women of reproductive age to measure changes in the contraceptive prevalence rate (CPR) and other family planning-related variables. At baseline in July 2010, 2,676 women were interviewed; about 50% were successfully tracked and interviewed at endline in December 2014. A baseline service delivery point (SDP) survey of 112 health facilities and 303 service providers was conducted in July 2011, and an endline SDP survey was conducted in December 2014 to measure facility-based interventions. The SDP baseline survey was conducted after the household survey, as facilities were selected based on where clients said they obtained services.

**Results:**

The project led to significant increases in use of implants and intrauterine devices (IUDs). Uptake of implants increased by 6.5 percentage points, from 2.4% at baseline to 8.9% by endline, and uptake of IUDs increased by 2.1 percentage points, from 2.2% to 4.3%. By the endline survey, 37.7% of clients using pills and injectables at baseline had switched to LARCs. Contraceptive use among the poorest and poor wealth quintiles increased by 20.5 and 21.5 percentage points, respectively, from baseline to endline. Various myths and misconceptions reported about family planning methods declined significantly between baseline and endline.

**Conclusion:**

Training, commodity security, multiple service delivery models, and demand promotion were the cornerstones of a successful approach to reach the urban poor in Nairobi with LARCs.

## Abstract (precis)

Use of long-acting reversible contraceptives increased significantly among women in a poor, urban setting through training, mentoring, commodity security, quality improvement, multiple service delivery models, and multiple demand-promotion approaches.

## BACKGROUND

Long-acting and reversible contraceptives (LARCs), comprising implants and intrauterine devices (IUD), offer immense potential to meet the need for family planning because they are safe, highly effective, and do not rely on adherence or postcoital vigilance, as is the case with pills, injectables, barrier methods, and emergency contraception. LARCs have a higher continuation rate and are more cost-effective than short-acting methods, and they do not have the health risks associated with estrogen-containing contraceptives.^1^ However, in many countries in sub-Saharan Africa, fewer than 5% of women who reported using contraception in the past decade were using LARCs.^2^ This proportion has not changed significantly. The reasons for low uptake include misperceptions about the safety and efficacy of LARCs, perceived lack of consumer demand, inadequately trained providers, and the relative complexity of providing LARCs compared with short-acting methods.^3^ In Kenya in 2008, only 1.1% of sexually active women used IUDs and 1.3% used implants.^4^

The reasons for low uptake of LARCs include misperceptions about their safety and efficacy, perceived lack of consumer demand, inadequately trained providers, and the relative complexity of providing LARCs.

To address the unmet need for family planning, especially among the urban poor, the Bill & Melinda Gates Foundation launched the Urban Reproductive Health Initiative (URHI) in India (Uttar Pradesh), Kenya, Nigeria, and Senegal. The project in Kenya, named Tupange for “Let’s plan” in Swahili, was implemented by a consortium of 5 agencies, led by Jhpiego, and including Marie Stopes Kenya, Pharm Access Africa Ltd, the National Council for Population and Development, and the Johns Hopkins Center for Communication Programs. The 5-year Tupange project was implemented in Kakamega, Kisumu, Machakos, Mombasa, and Nairobi. Tupange, in collaboration with the Ministry of Health (MOH), implemented demand- and supply-side interventions to increase the CPR. In Nairobi, we implemented the project in 92 public and private health facilities between July 2011 and December 2014, with a focus on poor urban areas.

Nairobi typifies the rapid urbanization and population explosion in sub-Saharan Africa. Nairobi’s population is 3.1 million, and it has an estimated annual population growth of 4.1%.^5^ A consequence of the rapid and uncontrolled population explosion is the proliferation of informal settlements, with an estimated 60% to 70% of residents living in slums.^6^ Tupange worked in all 9 subcounties in Nairobi.

## THE TUPANGE INTERVENTIONS

**FIGURE 1.**
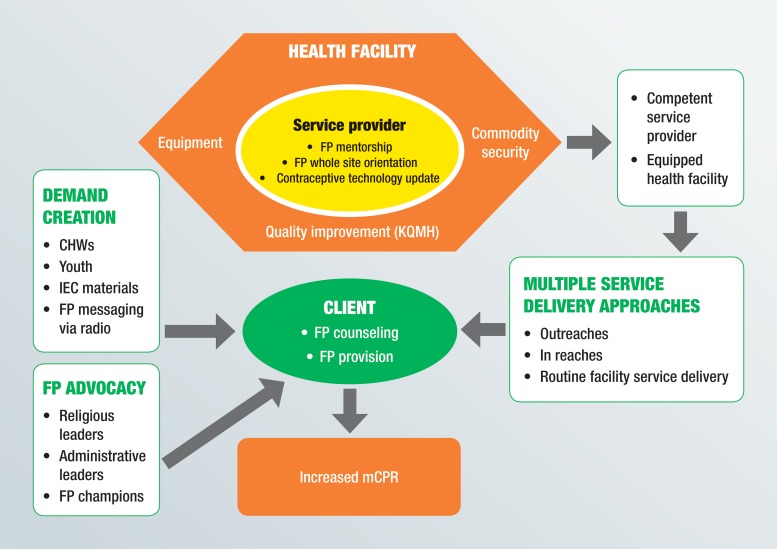
Tupange Project Conceptual Framework Abbreviations: CHW, community health worker; FP, family planning; IEC, information, education, and communication; KQMH, Kenya Quality Model for Health; mCPR, modern contraceptive prevalence rate.

Tupange’s interventions included building capacity of service providers, improving quality, increasing commodity security, establishing multiple demand-promotion and service delivery models, and conducting advocacy for family planning (Figure 1). The project provided technical support; training materials; medical consumables logistical support; and information, education, and communication materials.

### Capacity Building

A baseline service delivery point (SDP) survey in July 2011 showed that most service providers were not skilled to provide LARCs, and facilities were poorly equipped. The Tupange project addressed these challenges in a variety of ways.

#### Contraceptive Technology Updates

We provided all intervention sites with 5-day theory-based and practical trainings on basic family planning, focusing on short- and long-acting methods. Service providers practiced insertion skills on models and performed role plays to strengthen their counseling skills. Competency checklists were used to assess skills.

#### Mentoring

We offered facility-based training to service providers with a skilled mentor who gave step-by-step guidance on LARC services. Service providers needed to have basic family planning training to qualify for mentoring. The timing of the mentoring was dependent on the availability of the mentor and the service provider and the workload at the facility. Each service provider had to conduct 10 implant (Implanon or Jadelle) insertions and 10 copper IUD insertions as well as 5 implant and 5 IUD removals under the mentor’s observation before they were evaluated and certified by an external assessor. The service providers’ log books were used to track insertions and removals. To ensure objectivity, the assessors used the competency checklists for clinical assessment.

Each service provider had to conduct 10 implant and 10 IUD insertions as well as 5 implant and 5 IUD removals under the mentor’s observation before they were evaluated and certified by an external assessor.

Because mentors had different training backgrounds, we conducted a 2.5-day skills-standardization training. Mentoring involved a didactics session coupled with practice on models. Initially, subcounty reproductive health coordinators worked with facility mentors to ensure that they were mentoring according to the standard. Once the coordinators confirmed the quality of the mentoring, it became a purely facility-based activity.

Mentorship assessment was a challenge early in the project due to the limited number of clients visiting facilities for IUDs. Later, the coordinators conducted mentorship assessments during integrated community outreach events where health wagons created an ideal clinical setup. Health wagons are mobile clinics pulled by a truck with essential equipment and supplies for offering health services.

#### Whole-Site Orientation

We conducted whole-site orientations in health facilities to build the capacity of the staff. Both clinical and nonclinical staff (including cleaners and watchmen) received updates on family planning and LARCs through low-dose, high-frequency training. The orientation entailed 12 one-hour sessions at the facility at a convenient time that minimized disruption of services. In addition to covering family planning in general, the orientation focused on interpersonal communication skills, commodity management, and correcting family planning myths and misconceptions, especially about LARCs, among staff. Tupange used the provider-initiated family planning approach, which integrates family planning into other services to avoid missing opportunities to offer family planning. Staff were oriented to refer appropriate clients, who were visiting the facility for other reasons, to the family planning room for counseling and services.

Both clinical and nonclinical staff (including cleaners and watchmen) received updates on family planning and LARCs through low-dose, high-frequency training.

#### Equipment

The Tupange baseline survey and a facility rapid assessment in 2011 identified a lack of or inadequate essential LARC equipment and supplies. Tupange procured and distributed essential equipment and supplies on a need basis. These included implant and IUD insertion/removal sets, blood pressure machines, speculum sets, forceps, autoclaves, screens, examination couches, handwashing buckets, examination lights, Macintoshes (rubberized cloths used to cover examination couches for infection prevention purposes), decontamination buckets, and heavy-duty gloves. Equipment distribution was done in conjunction with the subcounty teams, and the MOH included the equipment in its inventory.

#### Kenya Quality Model for Health

It was difficult to uniformly assess the quality of services at supported facilities because they implemented different quality-improvement models. Therefore, the county government adopted the Ministry of Health’s Kenya Quality Model for Health (KQMH) as the universal model for monitoring quality improvement. KQMH involved formation of work improvement teams with representatives from all service delivery areas and the community served by the facility. The teams met regularly to assess the quality of services in all service delivery areas and to identify areas of improvement. Teams used the National Integrated Family Planning Standards to identify family planning gaps. Facilities that performed well served as benchmarks for other facilities to learn from.

#### Supportive Supervision

Tupange supported quarterly integrated, supportive supervision visits by the county and subcounty health management teams, who worked with service providers to address challenges faced by facilities in the provision of LARC services.

### Commodity Security

At the beginning of the project, commodities were available at the national stores, but challenges in last-mile logistics meant commodities were not routinely available at the point of use. Tupange’s innovative approach to addressing last-mile logistics included training of service providers and commodity managers on family planning commodity management through a 2.5-day module, use of an SMS (short message service, or text message) commodity tracking system, and redistribution of commodities based on real-time data from the SMS system (the Informed Push Model). The project included refresher and on-the-job trainings to address staff transfers and increase the number of staff competent in commodity management. Tupange also strengthened the national commodity supply pipeline by participating in the national technical working group on commodity security.

### Service Delivery Models

In addition to routine facility service delivery, Tupange used integrated outreach and in-reach activities to offer women family planning, including LARCs. Integrated outreach activities took place in hard-to-reach areas in the community to take services where they were needed. Integrated services during outreach activities included cervical cancer screening, HIV testing and counseling,deworming for children, growth monitoring, and vitamin A administration.

Integrated outreach activities took place in hard-to-reach areas in the community to take services where they were needed.

In-reach activities involved a team of a doctor, a nurse, and a care assistant who provided efficient, focused, high-quality services at high-volume facilities on scheduled days. They had equipment and supplies to offer LARCs and permanent methods (PMs). Community health workers conducted demand-promotion activities in advance while service providers at the facility booked clients. During the in-reach, the team provided LARCs and PMs while the facility staff provided short-acting methods and other routine services. Staff interested in LARC provision skills were mentored during in-reach activities.

### Multiple Demand-Promotion Activities

Tupange worked with community health workers, youth groups, religious leaders, national and county administrators, and local radio stations to inform communities about family planning.

#### Community Health Workers

Community health workers (CHWs) are guided by the community strategy outlined in the Kenya Essential Package for Health. Each subcounty is divided into several community units. A community unit is the lowest level of service delivery and is served by 50 CHWs and 2 community health extension workers (CHEWs) linked to a specific health facility. In Nairobi’s informal settlements, community units serve a population of more than 10,000 people—well above the MOH’s recommendations of about 5,000 people—due to overcrowding.

CHWs are lay health workers trained to deliver an intervention at the community level; they have no formal professional or paraprofessional certificate or tertiary education degree. Tupange trained CHWs in a 5-day family planning course to prepare them to deliver community-based services: health education, community-based distribution of condoms and pills, client referral, and data collection and documentation. To update CHWs and enhance facility–community linkage, facility in-charges and CHEWs conducted monthly supervisory meetings. CHWs were supported with working tools such as a flip chart of family planning methods, a bag for carrying family planning commodities, referral tools, umbrellas, and rain boots.

Apart from individual health education by CHWs, Tupange supported community dialogue days and action days when participants discussed family planning. Satisfied LARC users discussed their experiences with the methods and dispelled myths and misconceptions about family planning.

Satisfied LARC users discussed their experiences with the method and dispelled myths and misconceptions about family planning.

#### Youth Groups

Youth groups played a key role in demand promotion for family planning in Nairobi. They used edutainment and other activities to reach fellow youth and the general population: magnate theater (a form of interactive community theater that typically takes place in outdoor, public spaces); mini caravans (small vehicles mounted with a public address system and with actors to create demand for family planning in the community); acrobatic shows; posters; letters to churches and mosques; information, education, and communication materials; Miss Tupange beauty pageants; puppeteers; and football tournaments.

#### Religious Leaders

Religious leaders have long been considered a barrier to family planning use. In the Tupange project, however, religious leaders were allies and promoted family planning during religious services. They offered churches and mosques as venues for service delivery during integrated family planning outreach services. Religious leaders were oriented on family planning to dispel myths and misconceptions.

#### County and National Administrators

Tupange oriented county and national government administrators on family planning so that they could advocate family planning. Chiefs invited service providers during their *baraza* (public meeting) to inform participants about family planning.

#### Local Radio Stations

Tupange partnered with local radio stations to inform listeners about family planning through an award-winning radio drama series (*Jongo Love*), radio spots, and expert interviews. The project formed community radio drama listening groups to discuss issues arising from the radio drama series. Experts provided information on the radio about family planning, including LARCs, and dispelled myths and misconceptions. The radio stations announced Tupange-supported service delivery events, which led to high turnouts.

Table 1 and Table 2 summarize the Tupange activities and numbers of participants and clients.

**TABLE 1.** Participants in Tupange Activities, Nairobi, Kenya, July 2011–December 2014

Type of Activity	Number
Capacity building	
Service providers completing contraceptive technology updates	188
Service providers completing family planning mentoring	103
Staff receiving family planning whole-site orientation	538
Commodity security	
MOH staff trained in commodity management	168
Service delivery	
Family planning integrated outreach activities conducted	427
Family planning in-reach activities conducted	1,770
Demand promotion	
CHWs who worked with Tupange	630
Youth groups in Nairobi that worked with Tupange	9
Youth oriented on family planning and communication skills in Nairobi prior to being engaged for community outreach	220

Abbreviations: CHW, community health worker; MOH, Ministry of Health.

**TABLE 2.** Clients Reached by the Tupange Project, Nairobi, Kenya, July 2011–December 2014

Type of Activity	Number
Service delivery	
Family planning clients served through integrated outreach services (mobile sites)	52,557
Clients served during in-reach activities (fixed sites)	68,293
Clients served (new and revisits) in Tupange-supported facilities: facility, in-reach, and outreach	808,553
Community outreach	
Clients reached by CHWs and youth groups during community outreach	401,309
Referrals by CHWs to health facilities	67,447

Abbreviations: CHW, community health worker.

## METHODS

### Study Design and Sampling

URHI used a prospective cohort design with baseline and endline household surveys to assess changes in the CPR and other outcomes of interest resulting from the Tupange project. The Measurement, Learning & Evaluation (MLE) Project of URHI coordinated this rigorous evaluation. MLE is a partnership of the Carolina Population Center at the University of North Carolina, the African Population and Health Research Centre, and the International Center for Research on Women. In Kenya, MLE carried out the surveys through the Kenya Medical Research Institute and the Kenya National Bureau of Statistics. Institutional review boards in Kenya and the United States approved the surveys.

MLE also conducted SDP surveys at baseline and endline to present a holistic picture of the project, focusing on health facility factors. We analyzed project data collected during implementation to measure uptake of family planning services, including LARCs.

#### Household Surveys

MLE undertook a longitudinal survey to collect data on households and women at baseline (July 2010) and endline (December 2014) in the 5 project cities in Kenya.

At baseline, MLE used a 2-stage cluster sampling in the 5 cities. In Stage 1, MLE selected a random sample of clusters in each city. In Nairobi, 71 clusters were randomly selected and included both the formal settlements of the city and the informal settlements. The clusters were a representative sample of households identified using the 2009 Kenya population and housing census. In Stage 2, MLE selected a random sample of 30 households from each cluster.

A total of 4,260 households were selected for interviewing in Nairobi. The head of the household provided data on the household. Further, all women ages 15 to 49 years who were either residents or visitors present in the sampled household on the night before the survey were eligible for the women’s interview.

From the 4,260 households, MLE interviewed 2,676 women after they provided written consent. This set of women was tracked and interviewed at endline in 2014. Of the original cohort, 56.2%, representing 1,503 women, were tracked and found within Nairobi or within one of the study cities. At endline, 25.9% could not be tracked because their location could not be determined; 11.6% had moved from Nairobi to areas outside the study cities and were not tracked; 4.9% refused to participate; and 1.5% had died. Of the 1,503 women tracked at endline, 1,334 completed an interview.

We used 2 structured questionnaires to collect data; 1 for the head of the household and 1 for women in the households. Trained research assistants administered the household questionnaire, which assessed household assets and environmental circumstances, such as housing and sanitation. With this information, we developed a wealth index based on a principal component analysis of household assets. We used the wealth index to rank wealth status for each woman within a household. The research assistants also administered the women’s surveys, which collected information on demographic characteristics, current and past use of family planning, knowledge of family planning, and intention to use family planning.

MLE performed descriptive analyses of the women for selected independent variables including frequency distributions and percentages. The following independent variables were used within the study: age of respondent, education level, wealth index, number of live births, and marital status.

MLE stratified prevalence of contraceptive use by wealth quintile and contraceptive method, and compared the results from the baseline and endline periods. Differences in prevalence of contraceptive use at baseline and endline were measured for significance using the chi-square test of independence. Other analyzed variables included source of LARCs and specific statements on myths and misconceptions, and their prevalence compared at baseline and endline. All analysis presented are based on study weights.

Contraceptive prevalence was stratified by wealth quintile and contraceptive method, and comparisons were made between baseline and endline.

#### Service Delivery Point Survey

MLE conducted the baseline SDP study in July 2011 using a quasi-experimental longitudinal survey design; its main objective was to collect information on delivery of family planning services in Tupange cities. In Nairobi, the survey included 112 public and private health facilities, whether or not the Tupange project had supported them. The SDP survey collected 3 types of facility data: facility audit, service provider interviews, and client exit interviews.

MLE conducted a facility audit in all 112 facilities in Nairobi. The principal sources of data were the health facility manager and service statistics. The data collected included health services offered; types of family planning methods provided; human resource capacity to provide family planning services; availability of equipment and supplies for family planning services; and integration of family planning with other services, such as HIV, child immunization and growth-monitoring clinics, outpatient services, and maternal services, among others. Additionally, 4 service providers who routinely delivered family planning services were randomly sampled from each facility and interviewed. MLE interviewed a total of 303 service providers from the 112 facilities selected at baseline in Nairobi. The interviews collected data on providers’ ability to carry out family planning services, including their pre-service and in-service qualifications, the supervision they received, and their ability to integrate family planning services in the departments to which they were mainly assigned. MLE also conducted 1,397 interviews with female clients.

The endline SDP study, carried out in December 2014, aimed to determine the changes in availability of the various services at the end of the project. Because of the longitudinal nature of the study, all facilities surveyed at baseline were also surveyed at endline. In addition, the endline SDP survey included facilities that Tupange supported at endline but not at baseline, including new public health facilities. MLE surveyed and interviewed a total of 174 Tupange- and non-Tupange supported health facilities at endline. Of these, 35 Tupange-supported facilities had baseline and endline data and therefore are included in our analysis. At endline, similar to baseline, the survey included a facility audit, service provider interviews, and client exit interviews. Selection of service providers was random, and up to 4 service providers per facility were interviewed. MLE included for analysis all 128 service provider interviews at endline from the 35 Tupange-supported facilities. MLE obtained written consent before interviewing service providers and clients.

The variables of interest in the SDP survey were health facility participation in whole-site orientation, staff mentored on LARCs and PMs, staff trained on family planning commodity management, availability of written guidelines or service protocols for family planning services, existence of outreach programs, and availability of CHWs attached to the health facility.

The survey also assessed the status of family planning commodity stocks to determine if facilities had experienced any stock-outs within 1 year before the survey. Additionally, facility staff reported if they had the capacity to provide modern contraceptive methods and if they were offering the following modern methods: female sterilization, male sterilization, implants, IUDs, injectables, pills, emergency contraceptive pills, male condoms, female condoms, and the lactational amenorrhea method/breastfeeding. MLE used descriptive statistics to determine the prevalence of the variables under investigation.

## RESULTS

### Overview

The evaluation between baseline and endline involved a large number of participants assessed 4 years apart. Anticipating loss to follow–up, we ensured that there were adequate sample sizes at both time points to provide reliable estimates. Table 3 compares women interviewed at endline and women who took part in the baseline survey but were not interviewed at endline. The comparison shows similar background characteristics for the 2 groups in literacy and education. Women who were not interviewed at endline, however, were younger, and a higher percentage were never married, were Protestant, and had no children. We adjusted for these factors using study weights during subsequent analysis.

**TABLE 3.** Characteristics of Longitudinal Household Survey Respondents by Endline Status, Nairobi, Kenya, December 2014

	Not Interviewed at Endline (%) N=1,342	Interviewed at Endline (%) N=1,334	*P* Value
Age group			<.001*
15–19	12.5	7.4	
20–24	34.0	26.0	
25–29	25.6	24.4	
30–34	13.2	15.6	
35–39	8.5	13.3	
40–44	3.7	8.6	
45–49	2.4	4.7	
Education			.61
No education	2.5	2.0	
Primary incomplete	10.2	11.3	
Primary complete	26.6	27.2	
Secondary plus	60.7	59.5	
Wealth			<.001*
1 Poorest	19.4	16.3	
2 Poor	17.6	20.7	
3 Middle	20.3	18.2	
4 Rich	17.5	23.2	
5 Richest	25.2	21.7	
Religion			.02*
Protestant	67.9	68.0	
Catholic	25.1	25.6	
Muslim	5.7	3.8	
No religion	0.3	1.2	
Other	1.1	1.3	
Marital status			<.001*
Never married	40.2	29.6	
Married/living together	50.2	59.0	
Divorced/separated	8.2	8.5	
Widowed	1.4	3.0	
Literacy^a^			.49
Cannot read	2.4	1.4	
Can read only parts of sentences	17.1	16.0	
Can read whole sentences	78.5	81.4	
No card with required language	1.4	.6	
Number of live births			<.001*
No children	36.60	24.60	
1 child	31.40	24.70	
2 children	16.70	22.80	
3 children	8.80	13.60	
4 children	3.80	6.90	
5 children	1.40	4.10	
6+ children	1.20	3.40	

* *P*≤.05.

a Respondents were asked what language they were most capable of reading and given a card in that language with a sentence for them to read.

### Household Surveys

Table 4 presents background characteristics of the 1,334 women interviewed at baseline during the household survey and those who were tracked and interviewed at endline. At baseline, all women interviewed at their household were between 15 and 49 years of age. At endline, 3% of women interviewed were 50 years or older, and we did not include them in the CPR analysis.

**TABLE 4.** Women’s Background Characteristics at Baseline (July 2010) and Endline (December 2014), Nairobi, Kenya

	Baseline (%) N=2,676	Endline (%) N=1,334
Age group		
15–19	10.0	1.3
20–24	30.1	12.1
25–29	25.1	31.1
30–34	14.4	22.4
35–39	10.9	13.5
40–44	6.1	11.0
45–49	3.5	5.5
50–54	0.0	2.9
55–59	0.0	0.1
Education		
No education	2.3	1.9
Primary	37.6	33.6
Secondary	41.4	36.9
Higher than secondary	18.7	24.7
Nonstandard	0.0	2.6
Missing	0.0	0.3
Wealth		
Poorest	17.9	20.2
Poor	19.2	19.6
Middle	19.3	20.6
Rich	20.3	20.2
Richest	23.5	19.3
Number of live births		
No children	30.7	14.2
1 child	28.1	23.9
2 children	19.7	27.4
3 children	11.2	17.7
4 children	5.4	8.4
5 children	2.7	4.4
6+ children	2.3	4.1
Marital status		
Never married	34.9	21.3
Married/living together	54.3	60.1
Separated/divorced	8.3	14.4
Widowed	2.2	4.3

The educational status of women interviewed changed between baseline and endline. However, for both surveys, more than half of the women had a secondary education or above. The percentage of women with a primary or secondary education decreased slightly between baseline (41.4%) and endline (36.9%), whereas the percentage of women with a higher than secondary education increased. Additionally, the percentage of women with no education declined from 2.3% to 1.9% between baseline and endline.

At baseline, 30.7% of women had not had a live birth. This declined to 14.2% at endline. The percentage of women with 1–3 births increased by 10.0 percentage points, from 59.0% to 69.0%. Analysis by marital status showed that there was a decline in the percentage of women never married and an increase from baseline to endline among those married/living together or separated/divorced.

#### Contraceptive Prevalence Rate

There was a significant (*P*≤.05) increase in use of any contraceptive method among the poorest and poor quintiles, who were the project’s focus (Table 5). Among the poorest, the CPR for any method increased by 20.5 percentage points, from 41.7% to 62.2%, while among the poor it increased by 21.5 percentage points, from 47.9% to 69.4%. These increases in CPR among the poor and poorest were greater than those of the middle, rich, and richest quintiles, which recorded 5.1, 16.2, and 4.9 percentage point increases.

**TABLE 5.** CPR (%) by Type of Method and Wealth Quintile Between Baseline (July 2010) and Endline (December 2014), Nairobi, Kenya

	CPR Among All Respondents	CPR by Wealth Quintile				
		Poorest	Poor	Middle	Rich	Richest
Any method						
Baseline	47.8	41.7	47.9	57.2	47.5	44.9
Endline	61.6***	62.2***	69.4***	62.3	63.7***	49.8
Modern methods						
Baseline	43.6	37.6	44.6	53.8	42.5	40.1
Endline	54.8***	55.6***	64.0***	57.7	54.9***	40.9
Traditional methods						
Baseline	4.2	4.2	3.3	3.4	5.0	4.7
Endline	6.8**	6.6*	5.4	4.6	8.7	8.9*
No. of women						
Baseline	2,706	483	518	522	549	634
Endline	1,294	256	258	269	261	249

Abbreviation: CPR, contraceptive prevalence rate.

* *P*≤.05

** *P*≤.01

*** *P*≤.001.

**FIGURE 2.**
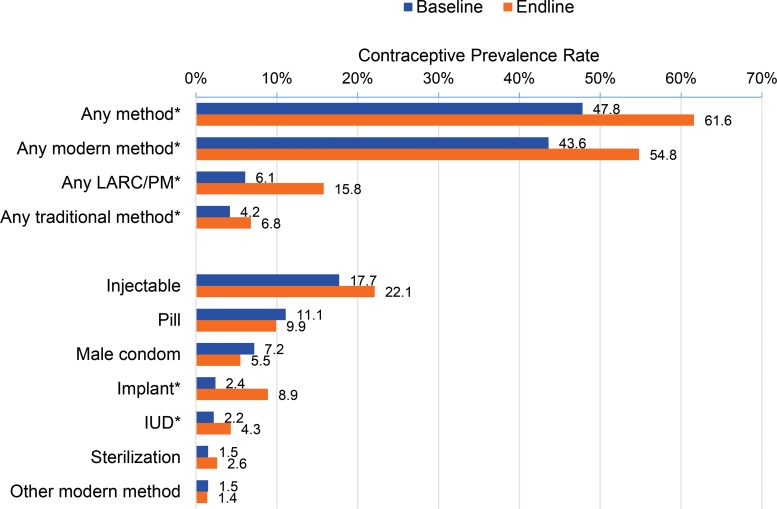
Comparison of Contraceptive Prevalence Rates at Baseline (July 2010) and Endline (December 2014), Nairboi, Kenya Abbreviations: IUD, intrauterine device; LARC, long‐acting reversible contraceptive; PM, permanent method. *P≤.05.

There was a significant (*P*≤.05) 6.5 percentage point increase in the uptake of implants, from 2.4% at baseline to 8.9% by endline, and a significant (*P*≤.05) 2.1 percentage point increase in the uptake of IUDs, from 2.2% to 4.3% (Figure 2).

Analysis of the CPR for any method by status of use at baseline and endline—to determine if a woman had switched to a different method—showed that 19.4% (n = 248) of women using injectables and 15.7% (n = 127) of women using pills at baseline had switched to a LARC. In addition, 7.2% (n = 698) of women who were not using any family planning method at baseline took up a LARC. Altogether, 10.2% (n = 1,329) of women who were not using a LARC at baseline were using a LARC at endline. There was an increase in the proportion of women reporting a public facility as their source of LARCs at endline compared with baseline, although it was not statistically significant (Table 6).

**TABLE 6.** Source^a^ of Modern Contraceptive Methods Among Women Between Baseline (July 2010) and Endline (December 2014), Nairobi, Kenya

Source	Baseline	Endline
Female sterilization^b^		
Public	NA	73.5%
Private	NA	26.5%
Other	NA	0.0%
Number	36^c^	34
Implant		
Public	48.4%	57.7%
Private	48.4%	36.9%
Other	3.1%	5.4%
Number	64	111
IUD		
Public	35.6%	45.5%
Private	64.4%	50.9%
Other	0.0%	3.6%
Number	59	55
Injectable		
Public	48.0%	38.8%*
Private	51.4%	61.2%*
Other	0.6%	0.0%
Number	477	273
Pill		
Public	29.8%	30.9%
Private	68.9%	68.3%
Other	1.3%	0.8%
Number	299	123
Male condom		
Public	10.5%	11.6%
Private	56.8%	49.3%
Other	32.6%	39.1%
Number	190	69

Abbreviations: IUD, intrauterine device; NA, not available.

a Public facilities include government hospital, government health center, and government dispensary. Private facilities include faith-based, mission hospital/clinic; private hospital/clinic; nursing/maternity home; community health worker/traditional birth attendant; traditional healer; pharmacy; and chemist. Other includes worksite clinic, mobile clinic, youth center, vending machine/dispenser, voluntary counseling testing/comprehensive care clinic, and bar.

b Bilateral tubal ligation.

c The 36 women reporting sterilization at baseline had missing responses on where they were sterilized.

* *P*≤.05.

Regarding myths and misconceptions, significantly fewer women agreed with any of the statements at endline than at baseline (Table 7). There was a reduction of at least 15 percentage points in common myths and misconceptions between baseline and endline.

**TABLE 7.** Percentage of Women Who Agreed With Statements About Family Planning Myths/Misconceptions at Baseline (July 2010) and Endline (December 2014), Nairobi, Kenya

	Baseline (%) N = 2,676	Endline (%) N = 1,334	Percentage Point Change
Can make a woman permanently infertile	53.7	27.4	26.3*
Users end up with health problems	75.4	48.4	27.0*
Can harm your womb	62.4	32.2	30.2*
Reduce woman’s sexual urge	63.1	46.1	17.0*
Can cause cancer	55.6	39.0	16.6*
Can give you deformed babies	63.9	26.2	37.7*
Are dangerous to your health	72.8	43.6	29.2*
Women who use them may become promiscuous	38.1	14.4	23.7*

* *P*≤.05.

### Service Delivery Point Surveys

Whole-site orientation on family planning took place in 88.6% of the selected Tupange facilities. More than half (70/128) of service providers interviewed at endline had undergone mentoring to improve their LARC and PM skills. The staff of 72.5% of supported facilities were trained on commodity management and reporting of commodity data.

At endline, about 85.7% of selected Tupange-supported facilities had outreach programs. All facilities conducting outreach activities reported discussing family planning/birth spacing and providing family planning services. Additionally, 85.7% of the selected Tupange-supported facilities reported a supportive supervision visit from Tupange teams for provision of LARCs and PMs. Nearly all (96.9%) of the selected Tupange-supported facilities had CHWs attached to them who were trained in family planning. Some of the demand-promotion strategies used by the selected Tupange-supported facilities during outreach events included CHW door-to-door visits (100.0%); youth group door-to-door visits (23.3%); loud speakers, drama, and puppetry (60.0%); and fliers and posters (56.7%). All the CHWs trained in family planning referred clients to the facilities for family planning services.

At baseline, 85.7% of the selected Tupange facilities offered IUDs and 82.9% offered implants, as shown on Table 8. At endline, 100% of facilities offered IUDs and implants. This 14-percentage-point increase for IUDs and the 17-percentage-point increase for implants are both statistically significant (*P*≤.05).

**TABLE 8.** Comparison of Selected Quality Outcomes From the SDP Survey of the Selected Tupange-Supported Health Facilities (N = 35) at Baseline (July 2010) and Endline (December 2014), Nairobi, Kenya

	Baseline (%)	Endline (%)
Facilities providing family planning services (by method)		
IUD	85.7	100.0*
Implants	82.9	100.0**
Any LARC/PM	88.6	100.0*
Facilities by number of modern methods provided		
No method	0	0
1–3 methods	0	0
4–6 methods	14.3	0
7+ methods	85.7	100.0*
Facilities with a stock-out in the last year (by method)		
IUD	20.0	0.0**
Implant	44.8	5.7***
Facilities with a stock-out in the last 30 days (by method)		
IUD	16.7	0.0*
Implant	17.2	0.0*

Abbreviations: IUD, intrauterine device; LARC, long-acting reversible contraceptive; PM, permanent method; SDP, service delivery point.

* *P*≤.05

** *P*≤.01

*** *P*≤.001.

At endline, all of the selected Tupange facilities had implants in stock, and only 5.7% had a stock-out in the year preceding the SDP survey. This is a significant change from baseline, when almost half of the selected facilities reported implant stock-outs in the year preceding the survey.

All of the selected Tupange facilities had IUDs in stock at endline, and none reported stock-outs in the past year. This was a significant (*P*≤.05) change from baseline, when 20% of the selected Tupange facilities reported stock-outs in the preceding year.

Client satisfaction was high at both baseline and endline; 97.0% of clients reported being satisfied with the family planning service they received. Additionally, 98.8% and 96.1% of women at baseline and endline, respectively, reported that they would use the health facility in the future, and 98.0% and 96.6%, respectively, said they would recommend the same facility to family, friends, and neighbors.

## DISCUSSION

The results support strategies at the health facility and in the community that can increase uptake of LARCs where they were previously either inaccessible or where the community had misconceptions about the methods. This multifaceted approach increased the uptake of LARCs among the urban poor, confirming previous findings from other countries.

Educating service providers and clients about the facts and benefits of LARCs and training them to offer LARCs is a key part of any strategy.^7^^,^^8^ Studies in South Africa and Zimbabwe reported that many clinicians have overly restrictive views of IUD candidates and unnecessarily limit access to the method.^9^ Inadequately trained staff is a key barrier to the provision of LARCs and other provider-dependent methods. Lack of sufficient contraceptive supplies complicates the ability of clinicians to offer LARCs,^10^ so Tupange educated health facility staff on commodity management. As the quality of services improved through the use of the KQMH, demand also increased as clients gained confidence in the staff.

Education is also important for prospective clients, as our study and others have shown. In Gabon, a study reported that most women in a postabortion ward had almost no knowledge of LARCs, but after counseling on LARCs and short-acting methods, nearly one-quarter left the facility with a LARC.^11^ Our study and others also provide evidence that linking demand promotion and service delivery can increase uptake of LARCs and other family planning methods.^3^

The results for Nairobi from the 2014 Kenya Demographic and Health Survey are in accord with our findings. Among all women in Nairobi, uptake of implants increased from 4.5% to 12.1%, and uptake of IUDs increased from 2.2% to 4.3%between 2008–2009 and 2014.^12^

### Limitations

This study had some limitations. First, the nearly 50% attrition rate over 4 years might have influenced some of the findings. However, we used appropriate weighting adjustments to take the attrition into account. Second, the reported increase in CPR between baseline and endline in all the cities cannot be directly attributed to the Tupange project alone, as other organizations and institutions also worked in Nairobi to strengthen family planning services. The study also may have been affected by recall bias.

## LESSONS LEARNED

Several facets of the Tupange project made key contributions to increasing use of LARCs among the poor urban population of Nairobi:

1. Training health care providers in implant and IUD services is critical. Cost-effective training models include contraceptive technology updates, facility-based mentoring, and whole-site orientation.
2. Health care providers should be trained to discuss LARCs with all women seeking contraception, including those interested in short-acting methods such as pills and injectables.
3. Common myths and misconceptions can be addressed by CHWs, youth groups, religious leaders, local leaders, and local radio stations.
4. The availability of family planning commodities at the national level does not necessarily lead to availability at service delivery points. Attention needs to be paid to last-mile logistics in the supply chain.
5. Improving the quality of family planning services can be achieved through a uniform quality-improvement model, reliable supplies of equipment and commodities, and continuous supportive supervision visits.

Common myths and misconceptions can be addressed by community health workers, youth groups, religious leaders, local leaders, and local radio stations.

We believe that elements of our successful project can be adapted and replicated in the public and private sector to produce long-lasting improvements in the uptake of LARCs across sub-Saharan Africa.

## Notes

**Peer Reviewed**

## References

[1] Speidel JJ Harper CC Shields WC. The potential of long-acting reversible contraception to decrease unintended pregnancy. Contraception. 2008;78(3):197–200. 10.1016/j.contraception.2008.06.001. 18692608

[2] Janowitz B Gmach R Otterness C The commercial sector’s role in providing long-acting and permanent methods. Bethesda (MD): Abt Associates, Private Sector Partnerships-One Project; 2006 Available from: http://pdf.usaid.gov/pdf_docs/Pnadh604.pdf

[3] Blumenthal PD Shah NM Jain K Saunders A Clemente C Lucas B Revitalizing long-acting reversible contraceptives in settings with high unmet need: a multicountry experience matching demand creation and service delivery. Contraception. 2013;87(2):170–175. 10.1016/j.contraception.2012.10.002. 23153895

[4] Kenya National Bureau of Statistics (KNBS); ICF Macro. Kenya demographic and health survey 2008-09. Calverton (MD): ICF Macro; 2010 Co-published by KNBS. Available from: http://dhsprogram.com/pubs/pdf/fr229/fr229.pdf

[5] Kenya National Bureau of Statistics (KNBS). The 2009 Kenya population and housing census. Nairobi (Kenya): KNBS; 2010 Available from: http://www.knbs.or.ke/index.php?option = com_phocadownload&view = category&id = 109:population-and-housing-census-2009&Itemid = 599

[6] African Population and Health Research Center (APHRC). Population and health dynamics in Nairobi’s informal settlements: report of the Nairobi cross-sectional slums survey (NCSS) 2012. Nairobi (Kenya): APHRC; 2014 Available from: http://aphrc.org/wp-content/uploads/2014/08/NCSS2-FINAL-Report.pdf

[7] Blumenthal PD Voedisch A Gemzell-Danielsson K Strategies to prevent unintended pregnancy: increasing use of long-acting reversible contraception. Hum Reprod Update. 2011;17(1):121–137. 10.1093/humupd/dmq026. 20634208

[8] Goldstuck ND Reducing barriers to the use of the intrauterine contraceptive device as a long acting reversible contraceptive. Afr J Reprod Health. 2014;18(4):15–25. 25854089

[9] Adjei KK Laar AK Narh CT Abdulai MA Newton S Owusu-Agyei S A comparative study on the availability of modern contraceptives in public and private health facilities in a peri-urban community in Ghana. Reprod Health. 2015;12:68. 10.1186/s12978-015-0058-z. 26253112 PMC4528711

[10] Morse J Chipato T Blanchard K Nhemachena T Ramjee G McCulloch C Provision of long-acting reversible contraception in HIV-prevalent countries: results from nationally representative surveys in southern Africa. BJOG. 2013;120(11):1386–1394. 10.1111/1471-0528.12290. 23721413 PMC3775997

[11] Mayi-Tsonga S Obiang PA Minkobame U Ngouafo D Ambounda N de Souza MH Introduction of postabortion contraception, prioritizing long-acting reversible contraceptives, in the principal maternity hospital of Gabon. Int J Gynaecol Obstet. 2014;126 Suppl 1 S45–S48. 10.1016/j.ijgo.2014.03.012. 24745694

[12] Kenya National Bureau of Statistics; Ministry of Health; National AIDS Control Council; Kenya Medical Research Institute; National Council for Population and Development; ICF International, The DHS Program. Kenya demographic and health survey 2014. Rockville (MD): ICF International; 2015 Available from: https://dhsprogram.com/pubs/pdf/FR308/FR308.pdf

